# Adenovirus F protein as a delivery vehicle for botulinum B

**DOI:** 10.1186/1471-2172-11-36

**Published:** 2010-07-07

**Authors:** Beata Clapp, Sarah Golden, Massimo Maddaloni, Herman F Staats, David W Pascual

**Affiliations:** 1 Veterinary Molecular Biology, Montana State University, Bozeman, MT 59717-3610, USA; 2 Department of Pathology, Duke University Medical Center, Durham, North Carolina 27710, USA

## Abstract

**Background:**

Immunization with recombinant carboxyl-terminal domain of the heavy chain (Hc domain) of botulinum neurotoxin (BoNT) stimulates protective immunity against native BoNT challenge. Most studies developing a botulism vaccine have focused on the whole Hc; however, since the principal protective epitopes are located within β-trefoil domain (Hcβtre), we hypothesize that immunization with the Hcβtre domain is sufficient to confer protective immunity. In addition, enhancing its uptake subsequent to nasal delivery prompted development of an alternative vaccine strategy, and we hypothesize that the addition of targeting moiety adenovirus 2 fiber protein (Ad2F) may enhance such uptake during vaccination.

**Results:**

The Hcβtre serotype B immunogen was genetically fused to Ad2F (Hcβtre/B-Ad2F), and its immunogenicity was tested in mice. In combination with the mucosal adjuvant, cholera toxin (CT), enhanced mucosal IgA and serum IgG Ab titers were induced by nasal Hcβtre-Ad2F relative to Hcβtre alone; however, similar Ab titers were obtained upon intramuscular immunization. These BoNT/B-specific Abs induced by nasal immunization were generally supported in large part by Th2 cells, as opposed to Hcβtre-immunized mice that showed more mixed Th1 and Th2 cells. Using a mouse neutralization assay, sera from animals immunized with Hcβtre and Hcβtre-Ad2F protected mice against 2.0 LD_50_.

**Conclusion:**

These results demonstrate that Hcβtre-based immunogens are highly immunogenic, especially when genetically fused to Ad2F, and Ad2F can be exploited as a vaccine delivery platform to the mucosa.

## Background

*Clostridium botulinum *is a Gram-positive, spore-forming anaerobe commonly found in soil, and is responsible for production of botulinum neurotoxin (BoNT), a potent lethal toxin [1]. Seven BoNT serotypes (A-G) have been identified [2]: serotypes A, B, E, and F are primarily responsible for human botulism; serotypes C and D are mostly limited to intoxication of animals [3]; and host specificity for serotype G has not been fully determined [4]. Three clinical manifestations are related to naturally acquired botulism: foodborne, infant, and wound botulism [5]. However, recently, three other forms have been described [6]: undefined or adult intestinal, inadvertent injection-related and inhalational botulism. All forms of BoNT intoxication present with the same clinical syndrome of symmetrical and descending flaccid paralysis of motor and autonomic nerves, which can cause respiratory arrest and death [7,8]. Since natural exposure to *C. botulinum *spores is rare, the necessity to create a vaccine is primarily for high-risk individuals. Moreover, an intentional release to contaminate food and water supplies or for use as an aerosolized weapon [3] warrants the development of vaccines to protect high-risk groups.

Currently, there are no certified vaccines for preventing human botulism. In the United States, an investigational pentavalent botulinum toxoid that includes serotypes A through E is used to vaccinate high risk individuals [9]. Although toxoid-based vaccines can be conveniently produced, these have a number of limitations, including isolation of active toxins, loss of essential neutralizing epitopes by formalin treatment, and multiple immunizations required to sustain elevated Ab titers [10]. To overcome these limitations, recent efforts have shifted to adapting recombinant BoNT heavy (H) chain to develop subunit vaccines [11,12], as well as employing DNA vaccine approaches [13,14]. Such approaches eliminate the reliance on handling active intact BoNTs.

BoNTs are originally produced as 150 kDa chains that are post-translationally nicked into a C-terminal H chain (ca. 100 kDa) and N-terminal light (L) chain (ca. 50 kDa) [13]. Functionally, the toxins contain three domains [15]: a receptor-binding domain, a translocation domain, and an enzymatic domain. Part of the receptor-binding domain is located in the carboxyl-terminal segment of the H chain, a region referred as the fragment C or Hc. The amino-terminal segment of the H chain is responsible for the translocation and internalization of the L chain into the cell [16]. The L chain acts as a zinc-dependent endoprotease, which selectively attacks three crucial proteins, disabling the docking and fusion of acetylcholine-containing synaptic vesicles to the plasma membrane [17].

A number of studies [rev. in [9]] have reported that recombinant 50 kDa Hc domain possesses the ability to stimulate protective Abs capable of neutralizing native BoNT. The Hc is an effective immunogen and simultaneously capable of binding and penetrating epithelial barriers in the gut and airway, suggesting that this molecule could be used as a mucosal vaccine against botulism [18]. The crystal structure of BoNT shows that the Hc fragment is composed of two subdomains: N-terminal developing jelly roll motif and a C-terminal forming β-trefoil domain [19,20]. The β-trefoil motif, especially the final approximately 150 residues, significantly diverges among serotypes with sequence similarity as low as 15% [21], but maintains a ganglioside binding motif that is conserved [22]. Since there is a lack of significant cross-reactivity among the serotypes, it is speculated that the protective epitopes are located within this fragment of Hc. In our previous study, vaccination with the Hc β-trefoil domain, referred to as Hcβtre/A, rather than the intact Hc, was shown to protect against BoNT/A [23].

A major impediment in forwarding mucosal vaccines into humans is the absence of mucosal adjuvants suitable for human use. Significant efforts have focused on live, attenuated vaccine approaches to circumvent the requirement for exogenous adjuvants. One such vaccine vector has been a modified adenovirus widely tested for a number of experimental vaccines [24]. A major benefit of adenoviral vectors is their affinity for the mucosal epithelium, which makes them optimal for mucosal vaccination [25], although this may be problematic for use in humans because of pre-existing Abs [26]. Initial attachment of adenovirus to the host cell surface via coxsackie-adenovirus receptor is mediated by the knob domain of adenovirus fiber [27]. To circumvent use of a live vector, the adenovirus 2 fiber (Ad2F) protein was selected and tested for its abilities to deliver BoNT/B Hcβtre vaccine. Previously, immunization with the fusion protein, Hcβtre/A-Ad2F, increased onset of plasma and mucosal Ab responses and conferred protection against BoNT/A [23]. In light of these findings, we queried whether this approach could be adapted to generate a vaccine for BoNT/B. Thus, Hcβtre/B was genetically fused to Ad2F, and immunization with Hcβtre/B-Ad2F was found to be more immunogenic than Hcβtre alone, further suggesting the utility of Ad2F as a targeting moiety to improve a mucosal vaccine's immunogenicity.

## Results

### Comparison of Ab responses by Hcβtre-or Hcβtre-Ad2F-vaccinated mice

**Figure 1:**
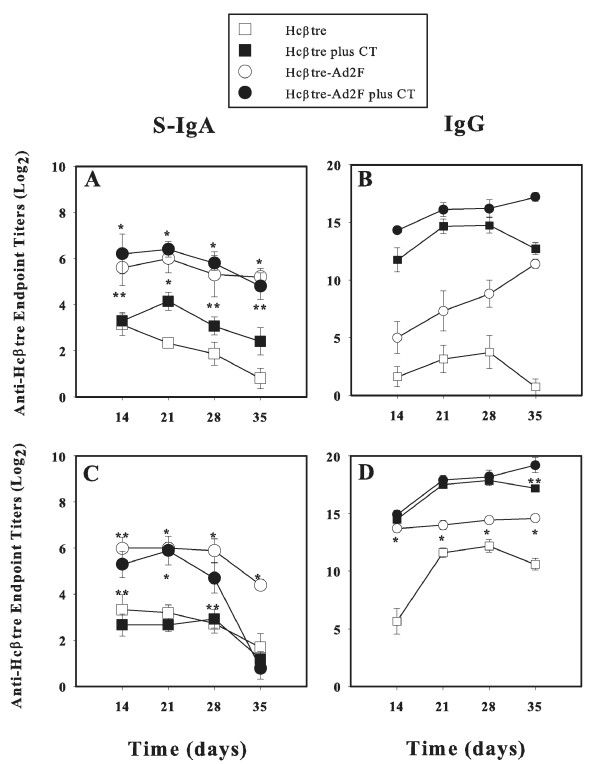
**Nasal or i.m. immunization with Hcβtre-Ad2F induces enhanced serum IgG and mucosal IgA anti-Hcβtre Ab responses**. BALB/c mice were immunized by the (A,B) i.n. or (C,D) i.m. route with equimolar concentrations of Hcβtre (25 μg) or Hcβtre-Ad2F (50 μg), either alone or with combination with cholera toxin (CT), as a mucosal adjuvant, on days 0, 7, and 14. Sera and fecal extracts were collected at weekly intervals beginning on day 14 post-primary immunization. Fecal IgA (A,C) and serum IgG (B,D) Ab titers against Hcβtre were determined by ELISA. The addition of vaccine-targeting molecule, Ad2F, in the absence and the presence of CT, improved mucosal Ab responses when compared to mice immunized with Hcβtre only. Without adjuvant, both groups induced weak IgG Abs responses. Data are expressed as mean ± SEM of three different experiments (n = 15 mice). *, p ≤ 0.001; **, p ≤ 0.05 represent the significant differences in IgA and IgG anti-Hcβtre levels between mice immunized with Hcβtre-Ad2F and Hcβtre with or without CT.

Groups of mice were immunized by the intranasal (i.n.) route with either Hcβtre or Hcβtre-Ad2F to assess the ability of each to evoke a mucosal and systemic immune response (Figure 1A,B). In parallel experiments, mice were given the same vaccines, but via the i.m. route (Figure 1C,D). All mice were boosted on days 7 and 14. Serum and fecal samples were collected at weekly intervals beginning on day 14 until day 35 post-primary immunization and then evaluated for anti-Hcβtre Ab titers by standard ELISA methods. Significant S-IgA Ab responses were detected in fecal extracts from mice dosed nasally with Hcβtre-Ad2F, either in the presence or absence of CT adjuvant, as opposed to mice dosed nasally with Hcβtre, which produced weak Hcβtre-specific S-IgA Abs (Figure1A). The intramuscular (i.m.) Hcβtre-Ad2F-immunized mice also exhibited elevated fecal Hcβtre-specific S-IgA Ab titers, but these tended to wane at the later time points (Figure 1C). In the presence of an adjuvant, i.n. (Figure 1B) and i.m. immunization (Figure 1D) with Hcβtre-Ad2F and Hcβtre induced elevated serum IgG Abs. Although mice i.n. immunized with Hcβtre-Ad2F alone or i.m. or i.n. with Hcβtre alone exhibited only weak serum IgG responses (Figure 1B,D), mice i.m. immunized with Hcβtre-Ad2F without CT showed modest induction of serum IgG Ab responses (Figure 1D).

**Figure 2:**
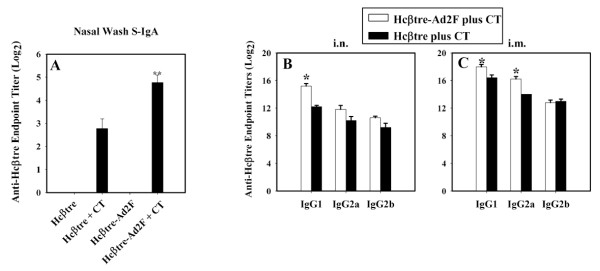
**Nasal immunization with Hcβtre-Ad2F plus CT stimulates an enhanced nasal wash secretory (S)-IgA and elevated serum IgG1 and IgG2a anti-Hcβtre Ab titers**. (A) BALB/c mice were nasally immunized with Hcβtre-Ad2F or Hcβtre plus CT, as described in Figure. 1. Three weeks after final immunization, S-IgA Ab levels in nasal washes were determined by ELISA. Mice vaccinated nasally with Hcβtre-Ad2F + CT showed significantly greater levels of Hcβtre-specific S-IgA when compared with mice nasally dosed with Hcβtre + CT. Data represent the mean ± SEM (n = 15 mice). *, p ≤ 0.001 represent the statistical differences in Hcβtre-specific S-IgA Abs between mice immunized with Hcβtre-Ad2F + CT vs. mice immunized with Hcβtre + CT. Serum IgG subclass anti-Hcβtre Ab responses on day 35 from (B) i.n. and (C) i.m. immunized mice (n = 7 mice/group) with Hcβtre-Ad2F or Hcβtre plus CT were evaluated. Significant elevations in (B,C) IgG1 and (C) IgG2a Ab titers were observed by the Hcβtre-Ad2F plus CT-immunized mice versus the Hcβtre plus CT-immunized mice: *, p ≤ 0.001.

To assess whether S-IgA anti-Hcβtre Abs were induced in nasal secretions, nasal wash samples collected on day 35 post-primary immunization showed significantly elevated S-IgA Abs in those mice immunized i.n. with Hcβtre-Ad2F plus CT when compared with mice given adjuvanted Hcβtre (Figure 2A). Mice dosed with the vaccines, but without adjuvant, showed no S-IgA Abs in their nasal washes (Figure 2A). Sera from the immunized mice were also evaluated for the induced IgG subclass responses from the CT co-immunized mice (Figure 2B,C). Both groups of i.n.-immunized mice showed elevations in their IgG1, IgG2a, and IgG2b anti-Hcβtre responses, but only the Hcβtre-Ad2F plus CT-immunized mice showed significant elevations in IgG1 responses (p ≤ 0.001) (Figure 2B). In contrast, the i.m. Hcβtre-Ad2F plus CT-immunized mice showed significant elevations (p ≤ 0.001) in both IgG1 and IgG2a anti-Hcβtre responses compared to the i.m. Hcβtre plus CT-immunized mice, and both groups showed equivalent IgG2b responses (Figure 2C). These results clearly show that Hcβtre evokes both mucosal and systemic immune Ab responses influenced by route of immunization, and the Ab titers are significantly improved when Hcβtre is genetically fused to Ad2F.

### Distribution of immune B cells following i.n. or i.m. immunization

**Figure 3:**
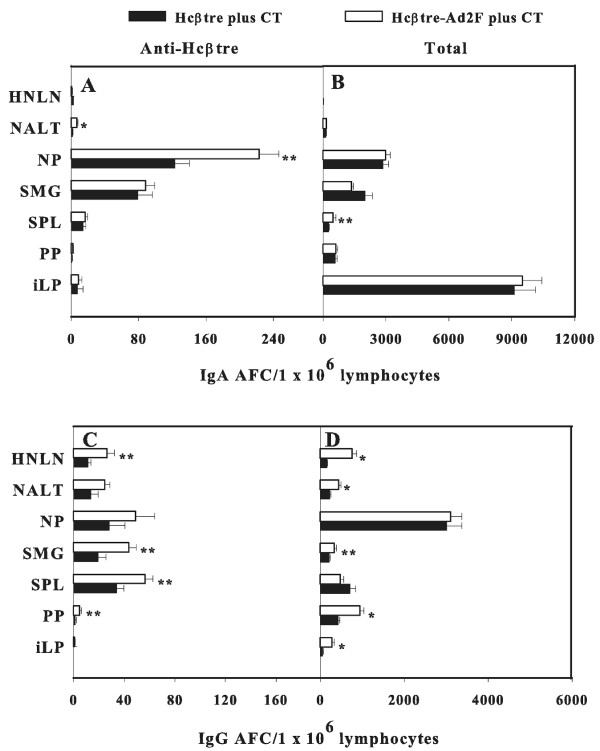
**Nasal immunization with Hcβtre-Ad2F plus CT enhances (A) IgA AFC responses in nasal passages (NPs) and NALT and (C) IgG AFC responses in head and neck LNs (HNLNs), submaxillary glands (SMGs), spleens, and Peyer's patches (PPs) when compared with Hcβtre plus CT**. Four weeks post-primary immunization, mononuclear cells from selected lymphoid tissues were evaluated by using B cell ELISPOT assay to determine the number and distribution of (A,C) Hcβtre-specific and total (B,D) IgG and IgG AFCs. Data are expressed as mean ± SEM of three separate experiments (n = 15 mice) of AFC/1 × 10^6 ^lymphocytes. *, p ≤ 0.001; **, p ≤ 0.05 represents the statistical differences in number of IgA and IgG AFCs in individual tissues between mice immunized with Hcβtre-Ad2F and Hcβtre with CT.

**Figure 4:**
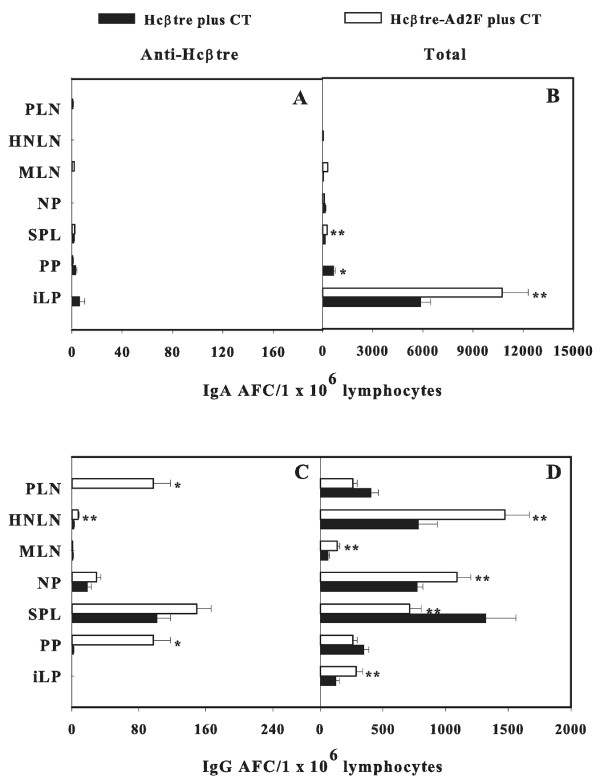
**I.m. immunization with Hcβtre-Ad2F plus CT enhances the number of IgG AFCs in peripheral LNs (PLNs) HNLNs, and PPs when compared to mice immunized with Hcβtre plus CT**. Four weeks post-primary immunization, mononuclear cells from selected lymphoid tissues were examined using B cell ELISPOT assay to determine the number and distribution of (A, C) Hcβtre-specific and (B, D) total IgA and IgG AFCs. Data are expressed as mean ± SEM of three separate experiments (n = 15 mice) of AFC/1 × 10^6 ^lymphocytes. *, p ≤ 0.001; **, p ≤ 0.05 represents the significant differences in number IgG AFCs in individual tissues between mice immunized with Hcβtre-Ad2F and Hcβtre with CT.

To learn the source of the immune Abs obtained upon immunization with Hcβtre-based vaccines, Ag-specific B cell responses from the various mucosal and systemic lymphoid tissues were measured using an Ab ELISPOT assay. Ag-specific Ab-forming cells (AFCs) were minimally detected in lymphoid tissues of mice vaccinated without CT (data not shown). Elevated Hcβtre-specific IgA AFCs were detected in nasal-associated lymphoreticular tissue (NALT) (p ≤ 0.001) and nasal passages (NPs) (p ≤ 0.05) from mice i.n. immunized with Hcβtre-Ad2F plus CT when compared to mice i.n. immunized with adjuvanted Hcβtre (Figure 3A). The numbers of Ag-specific IgA AFCs in head and neck LNs (HNLNs), submaxillary glands (SMGs), spleens, Peyer's patches (PPs), and intestinal lamina propria (iLP) were similar to mice immunized with either Hcβtre-Ad2F or Hcβtre (Figure 3A). Only minimal Ag-specific IgA AFCs were detected in mice i.m. immunized with our Hcβtre-based vaccines (Figure 4A). Analysis of Ag-specific IgG AFC responses in i.n.-dosed mice exhibited elevated AFCs in HNLNs, SMG, spleen, and PPs (p ≤ 0.05) of mice that received adjuvanted Hcβtre-Ad2F when compared with mice given Hcβtre (Figure 3C). The presence of Hcβtre-specific IgG AFCs was detected in NALT and NPs in both groups of i.n.-vaccinated mice, but these were not statistically significant (Figure 3C). Mice i.m. immunized with Hcβtre-Ad2F plus CT showed increased levels of Ag-specific IgG AFCs in the peripheral LNs (PLNs), PPs (p ≤ 0.001), and HNLNs (p ≤ 0.05) in contrast to mice that received Hcβtre plus CT (Figure 4C). Minimal IgA AFC responses were detected in any of the lymphoid tissues examined (Figure 4A). From these studies, we showed that nasal immunization with adjuvanted Hcβtre-Ad2F enhances IgA AFC responses in NP and NALT and IgG AFC responses in HNLNs, SMG, spleen, and PPs when compared with adjuvanted Hcβtre. In addition, these data show that i.m. immunization with Hcβtre-Ad2F in presence of CT enhances the number of IgG AFCs in PLNs, HNLNs, and PPs when compared to mice immunized with Hcβtre plus CT.

### Hcβtre-based vaccines elicit a mixed Th cell response

**Figure 5:**
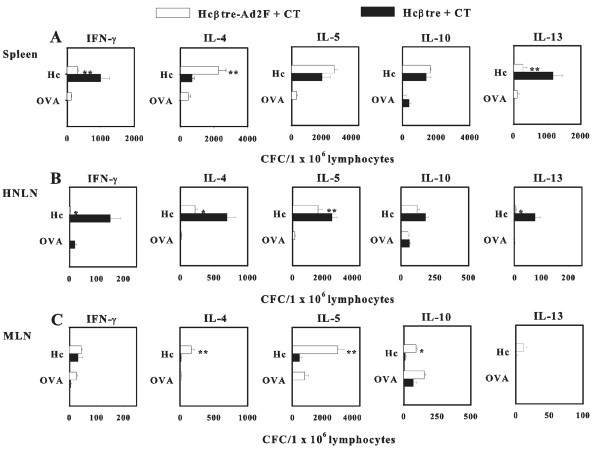
**Cytokine responses by i.n.-immunized mice with Hcβtre and Hcβtre-Ad2F plus CT show enhanced Th1-and Th2-type cell responses**. Three weeks after final immunization total lymphocytes from (A) spleens, (B) HNLNs, and (C) MLNs of Hcβtre plus CT-or Hcβtre-Ad2F plus CT-immunized mice were isolated and cultured with Hc/B, OVA, or media only for 2 days, and then CFC responses were measured by cytokine-specific ELISPOT. Immune lymphocytes were evaluated for IFN-γ, IL-4, IL-5, IL-10, and IL-13 CFCs/1 × 10^6 ^lymphocytes. Data represent the mean ± S.E.M. of three experiments (n = 15 mice). p ≤ 0.001; **, p ≤ 0.05.

**Figure 6:**
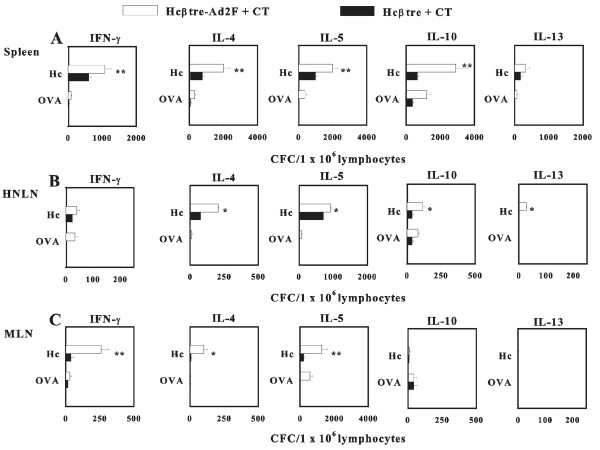
**Cytokine responses by i.m.-immunized mice with Hcβtre and Hcβtre-Ad2F plus CT show enhanced Th1-and Th2-type responses**. Three weeks after final immunization, total lymphocytes from (A) spleens, (B) HNLNs, and (C) MLNs of Hcβtre plus CT-or Hcβtre-Ad2F plus CT-immunized mice were isolated and cultured with Hc/B, OVA, or media only for 2 days, and then CFC responses were measured by cytokine-specific ELISPOT. Immune lymphocytes were evaluated for IFN-γ, IL-4, IL-5, IL-10, and IL-13 CFCs/1 × 10^6 ^lymphocytes. Data represent the mean ± S.E.M. of three experiments (n = 15 mice). p ≤ 0.001; **, p ≤ 0.05.

Since immunization with our Hcβtre-based vaccines induced elevated Ag-specific Ab responses in both mucosal and systemic tissues, it was important to examine the nature of Th cells that support these Hcβtre-specific B cell responses. Splenic, HNLN, and mesenteric LN (MLN) lymphocytes from mice dosed with adjuvanted Hcβtre and Hcβtre-Ad2F vaccines were restimulated with Hcβtre and subsequently evaluated for the production of IFN-γ, IL-4, IL-5, IL-10, and IL-13 by cytokine ELISPOT method. Analysis of Th1 and Th2 cytokine production by Ag-stimulated T cells revealed a varied Th cell phenotype. For i.n.-immunized mice, splenic IL-5 and IL-10 cytokine-forming cells (CFCs) were induced to similar levels for both vaccine groups, but increased IFN-γ and IL-13 CFCs were observed in Hcβtre-immunized mice and IL-4 in Hcβtre-Ad2F-dosed mice (Figure 5A). In the HNLNs, the IFN-γ, IL-4, IL-5, and IL-13 were particularly enhanced in Hcβtre-vaccinated mice (Figure 5B). In the MLNs, the levels of IL-4, IL-5, and IL-10 CFCs were significantly greater than in Hcβtre-Ad2F-dosed mice (Figure 5C). For i.m.-immunized mice, splenic IFN-γ, IL-4, IL-5, and IL-10 CFCs (Figure 6A), HNLN IL-4, IL-5, IL-10, and IL-13 CFCs (Figure 6B), and MLN IFN-γ,IL-4, and IL-5 CFCs (Figure 6C) were significantly elevated in the Hcβtre-Ad2F-immunized group when compared to Hcβtre-immunized mice. These results showed that nasal immunization with adjuvanted Hcβtre-Ad2F elicited an enhanced Th2 cell bias as opposed to mice immunized with Hcβtre plus CT that elicited a mixed Th cell response. I.m. immunization with either vaccine combined with adjuvant showed mixed Th cell responses. For either immunization route, it was evident that the IgG1 Ab responses were supported by elevations in Th2 cell cytokines and IgG2a Ab responses supported by IFN-γ.

### Mouse neutralization assay

To evaluate the protective Ab responses, serum samples from the various i.n. immunization groups were tested for their ability to neutralize native BoNT/B. Three sources of sera were assessed: (i) sera from unimmunized mice; (ii) sera from mice vaccinated with Hcβtre plus CT; and (iii) sera from mice vaccinated with Hcβtre-Ad2F plus CT. Mice were immunized four times on days 0, 7, 14, and 21, and serum samples were collected on day 57 post-primary immunization, at which time endpoint titers were determined for Hc/B. Mice given either Hcβtre or Hcβtre-Ad2F showed elevated serum IgG responses against Hc/B, while naive sera showed no reactivity (Table 1). Sera samples were incubated with 2.0 LD_50 _of the BoNT/B at room temperature for 60 min before i.p. injection into naïve BALB/c mice. All mice given naive sera succumbed to intoxication by 24 h (Table 1). Mice given BoNT/B plus pooled sera from mice nasally immunized with either adjuvanted Hcβtre or Hcβtre-Ad2F survived. These studies demonstrated that immunization with Hcβtre-Ad2F or Hcβtre could elicit neutralizing Abs that protect against systemic challenge.

**Table 1:** 24 h Survival status in BoNT/B-mouse neutralization assay^a^.

Vaccination Group^b^	Sera Titer^c^	%Survival^d^	p-value^e^
Naïve	0	0	-
Hcβtre/B + CT	19.5 ± 0.5	100	< 0.05

Hcβtre-Ad2F + CT	19.8 ± 0.2	100	< 0.05

^a ^Pooled sera from nasally vaccinated or naive mice incubated for 1 h with 2.0

LD_50 _of BoNT/B in PBS-0.2% gelatin and then injected into naive mice.

^b ^Source of sera used.

^c ^Endpoint titer for Hc/B in pooled sera (10 mice/group).

^d ^Survival of BoNT/B-challenged mice after 24 h.

^e ^p-value determined by Kaplan-Meier method.

## Discussion

Botulism results from absorption of the toxin into the systemic circulation from either a mucosal surface or a wound. BoNT intoxication is typically attributed to oral ingestion of contaminated food either with the toxin or bacteria producing the toxin [6]. Although natural exposure via the inhalational route is unlikely [18], illicit aerosol releases have occurred [28]; thus, there is a precedence to stimulate mucosal immune responses to aid in conferring protection by neutralizing the toxin at the mucosal surface. While most studies have focused on BoNT/A [13,29,30], few studies have evaluated BoNT/B-based vaccines [31,32]. Since BoNT/B is often implicated in human illness, the present study focuses on generating a protective mucosal vaccine.

Efforts to develop an efficacious vaccine for botulism are warranted because the current pentavalent toxoid vaccine has a number of disadvantages [23], including the limited ability to stimulate mucosal Abs when given parenterally [30] and the required yearly boosts to maintain protective Ab levels [13]. To address these shortcomings, recombinant vaccine approaches would best preserve neutralization epitopes, and consideration of mucosal immunization approaches may best serve to induce both mucosal and systemic immunity. Immunization of both mucosal and systemic immune compartments could then facilitate the clearance of the toxin from tissues and inhibit its absorption to mucosal surfaces [31,33].

Current efforts to develop BoNT vaccines have focused primarily on immunizing with recombinant Hc polypeptides capable of evoking protective immunity against BoNT challenge [10,12,34,35]. Hc is an efficient immunogen that combined with the ability to penetrate the epithelial membranes raises the possibility that the Hc can serve as a mucosal vaccine against botulism. Furthermore, as previously shown for protection against BoNT/A [23] and suggested by others [36], the entire Hc fragment is not required for protection and can be further reduced to contain the C-terminal β-trefoil subdomain. Our previous study provided the first evidence the Hcβtre fragment retains its immunogenicity and protective capacity when delivered nasally [23]. In addition, since β-trefoil structure is conserved among the seven BoNTs, it is plausible this conserved structure can evoke cross-reactive Abs against the other serotypes. In addition, we sought to develop a second animal model for BoNT intoxication using antisera developed in rabbits. When testing commercially prepared Hc against Hcβtre/A-Ad2F in rabbits formulated with CT or a non-toxin adjuvant, we found the Hcβtre/A-Ad2F vaccine was superior to Hc in conferring protection against BoNT/A challenge in a mouse neutralization assay when using antisera developed in rabbits (manuscript in preparation). These results suggest that the Hcβtre-Ad2F immunogen is effective in eliciting BoNT-specific Abs.

While next generation vaccines may significantly improve vaccine efficacy, these are often poorly immunogenic when applied mucosally [33]. To enhance their immunogenicity, vaccines would require an effective adjuvant to bolster protective immunity. Currently, a major obstacle to adapting mucosal vaccines for humans is the lack of a mucosal adjuvant suitable for use in humans. Nasal application of adenovirus-based vaccines offers one approach to stimulate robust systemic and mucosal Ab responses, as well as cell-mediated immunity [25]. In fact, recombinant adenovirus bearing the Hc transgene has been shown to effectively stimulate neutralizing Abs to BoNT/C [37]. However, one concern limiting the use of adenoviral vaccine vectors is that preexisting immunity to adenoviruses induced by natural exposure can potentially neutralize adenoviral vaccines via reactivity against its hexon protein [24], thus, preventing the development of a protective immune response [38]. Admittedly, studies have shown that i.n. immunization seems to avoid the stimulation of systemic neutralizing Abs [39]. However, another concern is the possible retrograde uptake of replication-deficient adenovirus vectors into the olfactory bulb [24], suggesting perhaps a natural consequence of wild-type Ad infection. Although adenoviruses have been found in the CNS, they are not generally considered as "neurotropic" and isolation of them from brain tissues is rare [40].

To circumvent such concerns, efforts were focused on developing a targeting moiety to enable mucosal vaccination based upon the adenovirus adhesin or F protein instead of the whole virus. In support of our previous study against BoNT/A [23], our current results clearly demonstrated that the inclusion of the targeting molecule, Ad2F, also boosts the levels of mucosal IgA and systemic IgG Abs against serotype B when administrated mucosally (Figure 1A,B). Moreover, it is important to emphasize that in the absence of CT adjuvant, nasal immunization with our recombinant Ad2F fusion vaccine stimulated similar levels of fecal IgA Abs as in mice co-immunized with CT (Figure 1A). In the absence of the Ad2F targeting moiety, Hcβtre induced only a weak mucosal Ab response even with CT. These findings suggest perhaps Ad2F possesses adjuvant-promoting properties, which, in turn, could limit the use of adjuvants and diminish the number of immunizations required to stimulate protective immunity. Current studies are addressing such possibilities. Interestingly, our study showed that mice given our fusion vaccine via the i.m. route produced elevated levels of both mucosal IgA and systemic IgG Abs (Figure 1C,D). Notably, i.m. immunization with Hcβtre-Ad2F in the absence of CT showed elevated IgG Ab titers that remained elevated for at least 35 days post-primary immunization. One possible explanation for our findings could be attributed to the interactions between Ad2F and the coxsackie adenovirus receptor, which is expressed in a wide range of cell types. This interaction may allow more effective delivery of the vaccine into the cells. In addition, similar to our previous study [23], the Hcβtre's immunogenicity was markedly improved by the addition of Ad2F (Figures 1, 2, 3 and 4). However, only a low level of mucosal Abs after day 35 was observed (Figure 1C), suggesting that long term mucosal memory was not induced when the Ad2F-based vaccine was given parenterally. Since very few IgA AFCs were detected in any of the lymphoid tissues of i.m.-immunized mice (Figure 4A), such evidence suggests that transient IgA Ab responses were induced during immunization.

Few studies have evaluated whether Th1 or Th2 cells are beneficial for optimal Ab production for the Hc-based vaccines. A recent study by Kobayashi et al. [30] suggests that BoNT/A-specific Abs induced by mucosal BoNToxoid immunization are mediated via Th2-type cytokines. Herein this report, a combination of Th cell types supports the BoNT/B-specific Ab responses (Figures 5 and 6). Notable enhancements in Th2-type cells were detected in the spleens and MLNs of i.n. Hcβtre-Ad2F plus CT-immunized mice (Figure 5A,C), and likewise for i.m. Hcβtre-Ad2F plus CT-immunized mice (Figure 6A,C) suggesting these responses supported the observed, elevated IgG1 Ab responses (Figure 2B,C). These findings further corroborate the results of our previous study showing that the Hcβtre/A vaccine for BoNT/A also elicits a mixed Th cell response [23].

Nasal administration of Hcβtre-based vaccines induced significant systemic and mucosal Abs. To assess their protective efficacy, a mouse neutralization assay, was performed (Table 1). Our data showed that passively administered sera from animals immunized with Hcβtre or with Hcβtre-Ad2F protected 100% mice for 24 hr against 2.0 LD_50_, suggesting neutralizing Abs are induced.

## Conclusion

As previously shown for Hcβtre/A [23], the Hcβtre/B is also very immunogenic capable of eliciting elevated serum IgG and mucosal IgA Abs, especially when genetically fused to the adhesin, Ad2F. These enhanced Ab responses are supported by mostly by Th2 cell responses when Hcβtre-Ad2F is given nasally, and a mixed Th1/Th2 cell response is obtained subsequent i.m. immunization. The induced Abs were able to neutralize BoNT/B as evident in the mouse neutralization assay. Thus, these studies demonstrate that the Hcβtre immunogen can be used as a BoNT vaccine.

## Methods

### Protein expression and purification

The strategy for the construction and expression of the recombinant proteins in *Pichia pastoris *was similar to those previously described [23]. A synthetic gene encoding for Hc/B amino acids E786 to E1291 (Genebank ID 6030102) was designed for expression *P. pastoris *taking into account the yeast *P. pastoris *taking into account the yeast codon bias, the reduction of *C. botulinum *A/T content, and the necessity for not depleting any particular tRNA pools. The Hcβtre/B spans from position E1071 to E1291, as predicted by others [22]. To clone the Hcβtre domain, we included seven more amino acids upstream from the predicted Hcβtre beginning with E1071 to E1291 to facilitate proper folding of the relevant domain.

To clone the Hc/B gene into the expression vector, the synthetic gene encoding for Hc/B was amplified with primers containing EcoRI and KpnI restriction sites. Likewise, to clone the Hcβtre, this gene was amplified from the synthetic Hc/B using primers containing EcoRI and KpnI restriction sites. In both cases, the 5' primers containing the EcoRI site also provided an ATG initiation codon embedded into an optimal Kozak's sequence. The PCR products were cloned into a pCR2.1 TOPO TA cloning vector (Invitrogen Corp., Carlsbad, CA), excised with EcoRI and KpnI, and cloned into the *P. pastoris *expression vector pPICZ B cut with EcoRI and KpnI. Such a vector was designed to provide a C-terminal histidine tag for subsequent protein purification. Ad2 fiber was amplified from genomic Ad2 DNA, as previously described [23], expressing only the C-terminal domain, G378 to E582, to include a short stretch of amino acids rich in Gly, the region containing the trimerizing domain and the region containing the globular knob domain, which is important for interacting with the coxsackievirus/adenovirus receptor on the cell surface [41].

To generate the Hcβtre/B-Ad2F, Hcβtre was amplified with primers generating EcoRI and SalI ends cloned and excised with EcoRI and SalI. The mentioned region of Ad2F was amplified with primer generating SalI and KpnI ends, cloned, and excised with the corresponding enzymes. The vector pPICZ B was cut with EcoRI and KpnI. Finally, Hcβtre-Ad2F was assembled *via *a tri-partite ligation. Primers were designed to allow in-frame junction between the different components and the formation of a flexible joint between Hcβtre and the Ad2F. These constructs were transformed into *P. pastoris *by electroporation.

Recombinant proteins in *P. pastoris *were purified following standard manufacturer's protocols. Following culture in YNB-methanol for 36-48 hr, cells were then harvested by centrifugation, and the pelleted biomass was disrupted with a bead-beater in ice. Debris was cleared by centrifugation, followed by filtration through a 1.2 μm pre filter, then by filtration through a 0.45 μm filter under vacuum. The cleared supernatant was then applied to a Talon column (BD Biosciences), as per the manufacturer's instruction. Purified proteins were eluted, titrated, and loaded onto a 12% polyacrylamide gel. Recombinant Hcβtre proteins were analyzed by Coomassie-stained SDS-PAGE to assess the quality of the protein.

### Immunization

BALB/c mice (National Cancer Institute) between 6 and 8 weeks old were maintained in the Montana State University Animal Resources Center under pathogenic free conditions in individually ventilated cages under HEPA-filtered barrier conditions; sterile food and water were provided ad libitum. The mice were free of bacterial and viral pathogens, as determined by Ab screening and histopathologic analysis of major organs and tissues. All animal care and procedures were in accordance with institutional policies for animal health and well-being and approved by MSU Institutional Animal Care and Use Committee.

Vaccines were administered by the intranasal (i.n.) or intramuscular (i.m.) route. For i.n. immunization, mice were given equimolar amounts of Ag corresponding to 25 μg of Hcβtre or 50 μg of Hcβtre-Ad2F and, in some instances as stated, given with 5 μg of cholera toxin (CT; List Biological Laboratories), as indicated, and boosted with their respective vaccines on days 7 and 14 after initial immunization with 2 μg of CT. For i.m. immunization, mice received 25 μg of Hcβtre or 50 μg of Hcβtre-Ad2F with or without 1 μg of CT on days 0, 7, and 14.

### Measurement of anti-Hcβtre Abs titers by ELISA

Endpoint Ab titers in serum, fecal extracts, and nasal washes generated to Hcβtre/B were determined by the standard ELISA methods [23]. Serum and fecal samples were collected weekly; nasal washes, on day 35 upon study termination, from each mouse were assessed for their Hcβtre-specific Ab activity. Fecal extractions and nasal washes were performed, as previously described [23]. Flat-bottom, 96-wells (MaxiSorb, Nunc) were coated with recombinant Hcβtre/B (5 μg/ml) and incubated at 4°C overnight, followed by washing with phosphate-buffered saline (PBS) containing 0.05% Tween 20 (wash buffer). The plates were blocked with 1% bovine serum albumin (BSA) in PBS for 1 h at 37°C. After washes, serial dilutions of serum or fecal samples in PBS containing 0.05% Tween 20 and 0.5% BSA were added to the plates and incubated at 4°C overnight. IgG, IgG subclasses, and IgA titers were determined using peroxidase-conjugated goat anti-mouse IgG, IgG1, IgG2a, IgG2b, or IgA (Southern Biotechnology Associates). Following 90 min of incubation at 37°C and the washing step, 2,2'-azinobis(3-ethylbenthiazoline-6-sulfonic acid; Moss) was added as a substrate, and the plates were incubated for an additional 60 min at 25°C. The endpoint titers were the reciprocal dilutions of the last dilution yielding an absorbance at OD_415 _above 0.100 OD units above negative controls.

### Lymphocyte isolation

Lymphocytes were isolated from nasal passages (NPs), submaxillary glands (SMGs), nasal-associated lymphoreticular tissue (NALT), small intestinal lamina propria (iLP), Peyer's patches (PPs), mesenteric lymph nodes (MLNs), spleens, peripheral LNs (PLNs), and head and neck LNs (HNLNs). PPs, MLNs, spleens, PLNs, NALT, and HNLNs were subjected to dounce homogenization, and resulting cell suspensions were filtered through Nitex (Fairview Fabrics), washed with complete medium (CM), and centrifuged. Mononuclear cells from NPs, SMGs, and iLP were isolated, as previously described [42]. Lymphocyte viability was > 95% from each tissue, as determined by trypan blue exclusion.

### Ab ELISPOT

The B cell ELISPOT assay was used to quantify the numbers of IgA and IgG Ag-specific Ab-forming cells (AFCs). Mixed cellulose ester membrane-bottomed microtiter plates (MultiScreen-HA; Millipore) were coated at 100 μl/well with sterile PBS containing 5 μg/ml of recombinant Hc/B. For total IgA or IgG AFC determinations, wells were coated with 5 μg/ml goat anti-mouse IgG or IgA Abs (Southern Biotechnology Associates) in sterile PBS and incubated overnight at 25°C. The plates were blocked with 200 μl/well of RPMI 1640 medium supplemented with 10% FBS (RPMI-FBS) for 1 h at 37°C. Blocking medium was discarded, and 100 μl of cells from each tissue at varying concentrations were added to each well. After incubation overnight at 37°C in a humidified chamber under 5% CO_2_, the plates were washed with PBS containing 0.05% Tween-20 followed by overnight incubation with 100 μl/well of 1.0 μg/ml HRP conjugates of goat anti-mouse IgG or IgA Abs (Southern Biotechnology Associates). Wells were washed, and substrate solution containing 3-amino-9-ethyl-carbazole was added. After 20-30 min, the reaction was stopped by rinsing water. AFCs were enumerated by counting using a dissection microscope (Leica).

### Cytokine ELISPOT

Total splenic, HNLN, and MLN mononuclear cells (5 × 10^6^/ml) were resuspended in CM and restimulated with 10 μg/ml recombinant Hc/B, OVA (tissue-culture grade; Sigma-Aldrich), or media in the presence of 10 U/ml human IL-2 (PeproTech) for 2 days at 37°C. Cells were washed and resuspended in CM. Stimulated lymphocytes were then evaluated by IFN-γ, IL-4, IL-5, IL-10, and IL-13-specific ELISPOT assays [23]. Cell suspensions were prepared at 2 different densities: 2.4 × 10^5 ^and 8 × 10^4 ^cells ml. ELISPOT plates (MultiScreen-HA; Millipore) were coated overnight with the desired anti-cytokine capture antibody at 25°C. Antibodies were removed, and plates were blocked with 200 μl/well of CM for 1 h at 37°C. Cell suspensions were added in triplicate to each well, and the plates were allowed to incubate at 37°C, 5% CO_2 _for 24 h. Plates were washed with PBS containing 0.05% Tween-20, and biotinylated antibodies for either IFN-γ, IL-4, IL-5, IL-10, or IL-13 (BD Pharmingen) were added, and plates were incubated overnight at 4°C. After washing the wells, plates were incubated for 1 h with a peroxidase-labeled anti-biotin Ab (Vector Laboratories) at 25°C. The plates were washed again and developed by incubating for 45 min with substrate solution containing 3-amino-9-ethyl-carbazole, followed by rinsing in tap water. Spots were counted using a dissection microscope (Leica).

### Mouse neutralization assay

For the mouse neutralization assay, groups of mice were immunized on days 0, 7, 14, and 21 with equimolar doses of Hcβtre or Hcβtre-Ad2F with CT. Another group was left unimmunized. Ab titers against Hcβtre/B and Hc/B were determined for all groups of mice. To assess the collected immune sera pooled from each vaccinated group, on day 57, 50 μl of pooled sera from each immunization group were diluted 1:2 in PBS containing 0.2% gelatin, incubated for 1 h at room temperature with 2.0 mouse LD_50 _of BoNT/B (1.0 × 10^8 ^MLD_50_/mg, Lot no. B041709-01; Metabiologics), and then injected i.p. into five naïve BALB/c mice. Mice were observed hourly for signs of BoNT intoxication, and when signs of neuromuscular weakness become obvious, animals were euthanized in accordance with AAALAC guidelines.

### Statistical Analysis

To evaluate differences among variations in Ab titers, Ab AFCs, and cytokine CFCs, an ANOVA followed by Tukey's method was used and discerned to the 95 percent confidence interval. The Kaplan-Meier method (GraphPad Prism, GraphPad Software, Inc., San Diego, CA) was applied to obtain the survival fractions following intoxication with BoNT/B. Using the Mantel-Haenszel log rank test, the P-value for statistical differences among surviving BoNT/B challenge from sera obtained from Hcβtre plus CT-or Hcβtre-Ad2F plus CT-vaccinated mice and naive mice were discerned at the 95 percent confidence interval.

## Abbreviations

Ad2F: adenovirus 2 fiber protein; AFC: Ab-forming cell; BoNT: botulinum neurotoxin; BSA: bovine serum albumin; CFC: cytokine-forming cell; CM: complete medium; CT: cholera toxin; Hc: carboxyl terminal domain of heavy chain; Hcβtre: β-trefoil domain of Hc for BoNT; HNLN: head and neck lymph node; iLP: intestinal lamina propria; i.n.: intranasal; i.m.: intramuscular; MLN: mesenteric lymph node; NALT: nasal-associated lymphoreticular tissue; NP: nasal passage; PLN: peripheral lymph node; PP: Peyer's patch; SMG: submaxillary gland.

## Competing interests

The authors declare that they have no competing interests.

## Authors' details

^1 ^Veterinary Molecular Biology, Montana State University, Bozeman, MT 59717, USA

^2 ^Department of Pathology, Duke University Medical Center, Durham, NC 27710, USA

## Authors' contributions

BC, MM, HFS, and DWP conceived and designed the experiments. BC performed the immune evaluation studies, and DWP conducted the mouse neutralization study. MM made the genetic constructs in yeast for Hc, Hcβtre, and Hcβtre-Ad2F, and SG purified the recombinant proteins. BC, HFS, and DWP analyzed the data. BC and DWP prepared the manuscript, and all authors read approved the final manuscript.
